# Assessment of Caregiving Burden of Family Caregivers of Advanced Cancer Patients and Their Satisfaction with the Dedicated Inpatient Palliative Care Provided to Their Patients: A Cross-Sectional Study from a Tertiary Care Centre in South Asia

**DOI:** 10.31557/APJCP.2021.22.7.2109

**Published:** 2021-07

**Authors:** Ajay Kumar Kondeti, Ambedkar Yadala, N. Rajya Lakshmi, C.S.K. Prakash, Gayatri Palat, Shoban Babu Varthya

**Affiliations:** 1 *Department of Radiotherapy and Oncology, Government General Hospital, Kurnool Medical College, Kurnool, Andhra Pradesh, India. *; 2 *Pain and Palliative Medicine, MNJ Institute of Oncology and RCC, Hyderabad, India, and the Director, PAX Asia Program, Two Worlds Cancer Collaboration, Canada. *; 3 *Department of Pharmacology, All India institute of medical sciences, Jodhpur, Rajasthan, India. *

**Keywords:** Cancer, palliative care, family caregivers, caregivering burden, satisfaction with palliative care services

## Abstract

**Background and objectives:**

Family Caregivers (FCs) of advanced cancer patients often suffer from caregiving burden due to stress arising from the responsibility of caregiving. During the course of their patients palliative therapy, FCs quality of life seems to be influenced by their satisfaction with the quality of patient care. In this study, caregiving burden of FCs and their satisfaction with dedicate Inpatient palliative care (IPC) services provided to their patients were studied.

**Material and methods:**

This cross-sectional study assessed 211 FCs of advanced cancer patients. Caregiving burden of FCs and their satisfaction with IPC were studied through Zarith Burden Interview (ZBI-12 version) and Family Carer Satisfaction with Palliative Care scale (FAMCARE-2) questionnaires, respectively. Descriptive and correlation analyses were deployed for data analysis.

**Results:**

The summative mean ZBI-12 score for FCs was 20.26±5.92, suggesting moderate to high caregiving burden among FCs. Significantly higher scores were observed among FCs who belonged to below poverty line (BPL) families(p=0.025), revealing higher caregiving burden among this lower income group. FCs who were male, unmarried, unemployed, and residing in rural experienced higher caregiving burden. However, it did not lead to a statistically significant difference. The summative mean FAMCARE-2 scale scores was 74.01±4.34, which suggested FCs high satisfaction with the palliative care services provided to their patients. FAMCARE-2 scale scores were lower for BPL families, but it was not statistically significant.

**Conclusion:**

FCs from lower-income groups experienced higher caregiving burden. It seems that IPC unit provided satisfactory services to advanced cancer patients, leading to enhancement of FCs satisfaction and consequently quality of life.

## Introduction

Cancer is one of the leading cause s of mortality and morbidity among the non-communicable diseases in South Asian countries, such as India. The subcontinent of South Asia hosts up to 25% of the total world population. Though separated by the geographical boundaries, the South Asian countries still share similar food habits, culture as well as life styles, resulting in prevalence of similar risk factors for cancer (Moore et al., 2010).

Thus, despite massive diversity across the region, there are significant similarities among these counties in terms of cancer incidence and prevalence as well as patient care services, including preventive, curative, and palliative care measures (Moore et al., 2010). Hence, similar strategies for comprehensive cancer management, including palliative care services, can be adopted for the whole region .

According to Global Cancer estimates (GLOBOCON 2020), the cancer burden will increase by 64.7% from 2020 to 2040 in medium HDI regions, such as South Asia (Sung H et al., 2020).In India like other South Asian countries, the cause of cancer is multi factorial, burden of cancer is multidimensional, and hence treatment requires multidisciplinary approch (Taneja et al., 2011). 

In India, more than 50 percent of cancer patients consult the doctor only at the terminal stages of their disease. As the disease initially does not present itself, rural India suffers the most as at least 70-80 percent of patients do not approach hospitals even at the end stage of the disease (Jacob et al., 2019). In advanced cancer patients, palliative care, along with curative therapy, plays a vital role in managing their disease.

Cancer is a chronic disease, which usually lasts for one to few years and requires long term medical care. It also restricts the daily activities of sufferers. Thus, it brings about considerable changes, physically, mentally, socially as well as spiritually, in the lives of both cancer patients and their family caregivers (FCs).

FCs are relatives, friends, or neighbours who aid the patients without being paid (Reinhard et al., 2008). FCs play a vital role in ensuring proper health care to their advanced cancer patients, thus impacting the patient’s course of disease progression, survival, and quality of life. This often results in psychological burden on both patients and FCs. They tend to suffer in relation to each other (Grunfeld et al., 2004). 

FCs are informal caregivers and differ from formal caregivers (who are paid for their services) in many aspects. FCs has little or no formal training in caregiving. In addition, they have additional responsibilities, such as treating patients’ minor symptoms and side effects at their homes, booking hospital visits, providing transport facilities to hospital, etc. (Kurtz et al., 2005). 

In some FCs, these factors may affect their regular life cycle. On the other hand, FCs may be emotionally unprepared for caring (Girgis et al., 2009). With progression of disease, patients’ dependency on their FCs increases (Delalibera et al., 2015).

The South Asian countries, such as India, are middle income countries in where there are limited care services . Hence, the focus of care services is mostly on the curative and to some extent on palliative care of the advanced cancer patients. Hence, in this study, we reviewed previous studies on caregiving burden of FCs 

With huge geographic and demographic diversity among Indian population, there is few studies investigated caregiving burden in Indian FCs and mostly were dine in urban areas (Lukhmana et al., 2015; Daya et al., 2018). We established a dedicated inpatient palliative care (IPC) unit in 2019 for providing inpatient palliative care services to advanced cancer patients. Quality of life in FCs seems to be influenced by their satisfaction with the quality of patient care provided in these kind of centres (Lee et al., 2016, Ullrich et al., 2017). Understanding of FCs concerns and needs, providing supportive care, and integrating FCs in the treatment and care planning represent important characteristics of palliative care (Hudson P et al.,2011).

However, in South Asian countries like India, lack of adequate literature addressing the satisfaction of FCs with palliative care resulted in lack of both quantitative as well as qualitative assessment of palliative care units. Hence, the objective of this study was to assess the caregiver burden and satisfaction of the FCs of advanced cancer patients with the palliative care services provided in a dedicated IPC unit of a tertiary care centre in India, South Asia . 

## Materials and Methods

*Study institution*

The Government general hospital (GGH), Kurnool Medical College, Kurnool, Andhra Pradesh, India, is a major referral tertiary care teaching hospital. About 10 million people utilize various specialty and subspecialty health care services of this hospital. The Department of radiotherapy and oncology of GGH is providing radiotherapy, chemotherapy as well as pain relief and palliative care services.

We established IPC unit in 2019 for providing specialized palliative care services to the terminally ill cancer patients , as part of comprehensive cancer management. We are providing palliative care through inpatient, outpatient, and home care-based services.

IPC unit has interdisciplinary team of a doctor, nurses, a counsellor, social workers, and volunteers with mandatory training in specialized palliative care. In addition, this unit is providing physical, psychological, psychosocial, spiritual, existential symptom relief, and end of life care services to advanced cancer patients, which are not provided in the regular inpatient care. 

In addition, we are providing other services to the FCs, such as counseling, empowerment of the family in taking care of the patient, caregiver support as well as conduction of regular caregiver meetings, to raise their awareness on palliative care.

*Study design*

This study was a hospital-based cross-sectional one, which included FCs of advanced cancer patients referred to our IPC unit. Eligible FCs were enrolled from March 2020 to November 2020. Enrolment of the FCs for the study was done within one week after their patient’s admission. Ethics Committee of Kurnool medical college, granted the necessary permission to conduct this study (KMC-IEC No. 110/2020).

*Sample size*

The sample size was calculated based on a previous study done by Kim Y et al. in which the proportion of caregivers with high burden was found to be 67.3% (Kim et al., 2008). Hence, with 95% confidence interval and assuming 10% non-response rate, we considered a sample size of 200 . 

*Inclusion criteria*

1. Unpaid FCs of terminally ill cancer patients

2. Aging more than 18 years 

3. Being able to communicate in either vernacular language (Telugu) or English. 

FCs who were primarily assisting patients at home were given preference. FCs were enrolled in the study after providing written informed consent

*Exclusion criteria*

1. Being unwilling to give informed consent

2. Not being able to communicate verbally or orally 

Up to 263 FCs of terminally ill cancer patients were screened based on the inclusion criteria. Out of them, 232 FCs were deemed eligible. Ten FCs were found to be ineligible by applying the eligibility criteria . Five FCs opted out of the study due to time constraints. Out of the remaining 217 FCs, six did not completed the interview, resulting in a total of 211 FCs. Data were collected through questionnaires which were complete either by the FCs or by trained interviewers.

*Data collection tools*

To assess FCs caregiver burden and satisfaction with palliative care provided to their relatives, FCs completed the following questionnaires .

1. Zarit Caregiver Burden Interview Short Form (ZBI-12): ZBI-12 is the short version of ZBI and consists of 12 items in two domains, namely personal strain (3 items) and role strain (9 items). Each question is scored through five-point Likert scale ranging from 0 to 4 (never to almost always). High score represent higher feel of burden. The following score range depicts the level of burden: 0 to 10 none to mild burden, 11 to 20 moderate burden, and above 20 high burden. This instrument has excellent psychometric properties and good internal internal consistency, correlation, and validity (Bedard et al., 2001).

2. Family Carer Satisfaction with Palliative Care scale (FAMCARE-2): FAMCARE-2 scale is a revised version of the FAMCARE tool used to measure family satisfaction with advanced cancer care, especially in inpatient settings. It consists of 17 items scored on a five-point Likert scale ranging from “very satisfied” to “very dissatisfied”. Higher scores denote better satisfaction with palliative care services among FCs. It includes four subscales, namely the management of physical symptoms and comfort (items), provision of information (4 items), family support (4 items), and patient psychological care (4 items)) (Kristjanson et al., 1993; Aoun et al., 2010). 

The vernacular (Telugu) language versions of ZBI-12 and FAMCARE-2 questionnaires were pretested for translational validity through forward and backward translation.

*Statistical analysis*

Descriptive analyses were applied to evaluate baseline demographics. Student t-test or One- way ANOVA was applied for analysing the difference between ZBI-12 and FAMCARE-2 scale scores regarding demographic variables. Multivariate analysis was performed to analyse the associations between demographic variables and mean ZBI-12 and FAMCARE-2 scale scores. All significance tests were two-tailed using a significance level of p< 0.05. Data analysis was done using SPSS Version 23 (IBM Corp. Released 2015. IBM SPSS Statistics for Windows, Version 23.0. Armonk, NY: IBM Corp.).

## Results

**Figure 1:**
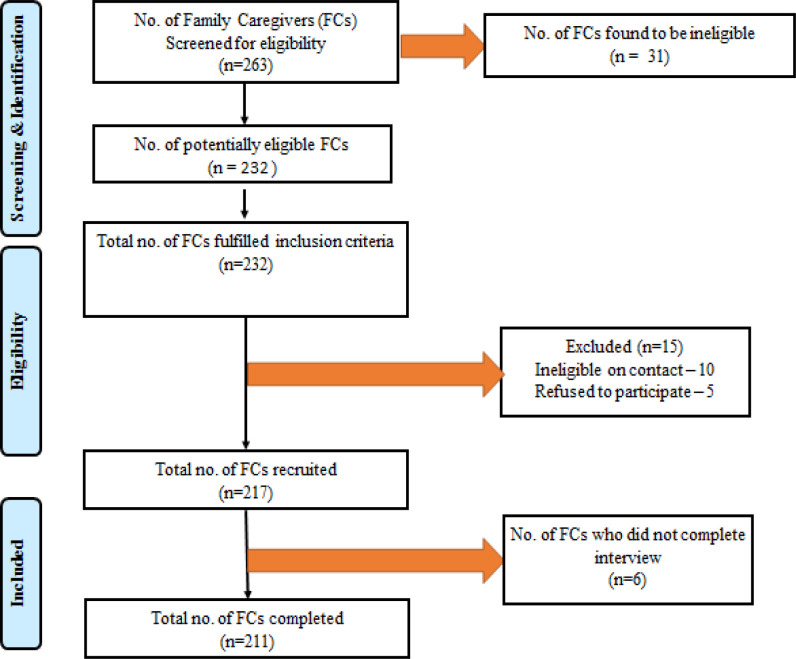
Family Caregivers (FCs) enrolment Flow Diagram

This study was conducted among FCs of advanced cancer patients admitted to IPC unit at the radiotherapy and oncology department of GGH. In this study, a total of 263 FCs were screened with respect to eligibility criteria . Among 263 FCs, only 232 were deemed eligible and 31 were ineligible. Among eligible FCs, 15 were excluded due to various reasons and 6 were lost to follow-up . Finally, 211 FCs completed this study. Flow diagram of enrolment is depicted in Figure 1.

The results showed that 59.7% of FCs were less than 60 years of age and the mean age of the FCs was 51.5 years (SD 14.20 and ranging from 19 to 74). Most of the FCs were male (52.1%), non-spousal caregivers (61.6%), and married (91%). Most of them belonged to majority Hindu religion (64.9%) and non-general backward communities (60.2%). Majority of them were literate (53.1%), employed (77.7%), below poverty line (BPL) families (69.7%), and from rural regions (78.7%). Head and neck cancers constitute majority of cases (41.2%) diagnosed in their relatives as shown in Table 1.

The summative mean ZBI-12 score for FCs was 20.26±5.92 (SD), suggesting moderate to high caregiving burden among FCs. The least score was 9; whereas, the highest burden score was 35 out of the maximum score of 40. Out of the total 211 recruited FCs, most of the FCs (51.65%) experienced moderate caregiving burden. About 44.07% FCs experienced higher caregiving burden; whereas, 4.26% FCs experienced only mild caregiving burden. Details of FCs ZBI-12 burden scores are summarized in Table 2. Statistically significant higher ZBI-12 scores were observed for FCs who belonged to below poverty line BPL families (p=0.025), suggesting higher caregiving burden among this lower income group. FCs who were male (p=0.382), unmarried (p=0.105), unemployed (p=0.161), and rural residents (p=0.326) had higher caregiving burden, but it was not significant. No significant association was observed among other socio-demographic characteristics regarding ZBI-12 scores (Table 1).

The summative mean FAMCARE-2 scale score was 74.01±4.34 (SD). Out of the maximum possible score of 85, our total mean scores suggested high satisfaction with the palliative care services among FCs . The minimum score observed was 57 and the maximum score was 81. 

Higher scores were also observed for all the subscales of FAMCARE-2 scale. Among the subscales, the highest mean score was allocated to subscale of management of physical symptoms and comfort, with overall mean of 20.93±1.69 (SD). The subscale of provision of information scored 17.54±1.13 (SD), subscale of family support scored 17.65±1.30 (SD), and the subscale of patient psychological care scored 17.78±1.44 (SD) (Table 3). 

FAMCARE-2 scores were lower in lower income group, that is BPL families, but it was not statistically significant (p=0.138). No significant association was observed among the other FC groups, regarding FAMCARE-2 scores, suggesting generally high level of satisfaction with palliative care provision among the FCs irrespective of their demographic characteristics (Table 1).

Multivariable regression results showed no significant association between FAMCARE-2 scores and demographic variables, such as age, sex, education level, income, and social status with respect to the satisfaction level (Table 4).

**Table 1:** FCs Variables, FCs Caregiving Burden, and FCs Satisfaction with Care

Variables FCs	Frequencies of FCs	ZBI-12 index		FAMCARE-2	
	(n=211)	Means (±SD)	p- value	Means (±SD)	p-value
1.Age groups					
60 years or older of age	85 (40.3%)	20.56±5.86	0.542	73.64±4.91	0.291
Below 60 years of age	126 (59.7%)	20.06±5.97		74.27±3.90	
2.Gender					
Male	110 (52.1%)	20.42±5.85	0.382	73.81±4.33	0.516
Female	100 (47.9%)	20.14±6.02		74.23±4.37	
3.Relation to the patient					
Spouse	81 (38.4%)	19.98±5.94	0.582	74.41±4.41	0.301
Non spouse	130 (61.6%)	20.44±5.92		73.77±4.29	
4.Marital status					
Unmarried	19 (9%)	20.47±5.87	0.105	74.63±4.27	0.512
Married	192 (91%)	18.16±6.12		73.95±4.35	
5.Religion					
Hindu	138 (64.9%)	20.38±6.18	0.917	73.93±4.43	0.975
Minority	73 (35.1%)	20.04±5.43		74.16±4.19	
6.Caste					
General	83 (39.3%)	21.06±5.50	0.554	73.98±3.69	0.878
Non-general	127 (60.2%)	19.82±6.10		74.03±4.74	
7.Education					
Illiterate	99 (46.9%)	20.01±5.97	0.565	74.16±4.59	0.644
Literate	112 (53.1%)	20.48±5.89		73.88±4.12	
8.Employment					
Unemployed	47 (22.3%)	20.57±6.04	0.161	74.13±4.26	0.84
Employed	164 (77.7%)	19.19±5.38		73.98±4.37	
9.Income					
BPL family	147 (69.7%)	20.86±6.67	.025*	73.72±4.61	0.138
APL family	64 (30.3%)	18.88±3.28		74.69±3.44	
10.Residency					
Rural	166 (78.7%)	20.47±6.27	0.326	74.10±4.19	0.599
Urban	45 (21.3%)	19.49±4.33		73.71±4.89	
11.Diagnosis of the relative					
Ca Head & Neck	87 (41.2%)				
Ca Breast	29 (13.7%)				
Gynaecological cancers	25 (11.8%)				
Ca Lung	13 (6.2%)				
GI malignancies	37 (17.5%)				
Other sites	20 (9.5%)				

Non General castes include OBC’s-other backward castes, SC-Scheduled castes and ST-Scheduled tribes; BPL family, Below poverty line family and APL family; Above poverty line family; *A clinically meaningful difference was observed

**Table 2:** ZBI-12 Burden Scores

ZBI-12 score	n =211	Percentage (%)
No-Mild burden (0-10)	9	4.26%
Moderate burden (11-20)	109	51.65%
High burden (>20)	93	44.07%

The summative mean ZBI-12 score was 20.26±5.92 (SD)

**Table 3:** FAMCARE-2 and Its Subscale Scores (n=211)

FAMCARE-2 Subscales	Means & SD scores
1.Management of Physical symptoms and comfort	20.93±1.69
2.Provision of Information	17.54±1.13
3.Family support	17.65±1.30
4.Patient psychological care	17.78±1.44
Total FAMCARE-2 score	74.01±4.34

**Table 4:** Multivariate Analysis of Association of FAMCARE-2 and ZBI-12 Scores along with Demographic Variables

Variables	OR	95%CI	p-value
ZBI-12	-0.32	-0.33 to -0.136	<0.001
Gender	0.05	-0.712 to 1.638	0.438
Age	-0.001	-0.041 to 0.040	0.990
Education	-0.006	-1.222 to 1.115	0.928
Income	-0.013	-1.619 to 1.376	0.873
Socioeconomic status	0.041	-0.508 to 0.909	0.578

## Discussion

Due to blood and brotherhood relations and financial constraints, FCs were left with no choice but to take care of their diseased relatives all by themselves (Yun et al., 2010). Limited availability as well as costly formal caregiving services further burdened the FCs in South Asian countries like India. Thus, Indian FCs face twin challenges of day-to-day livelihood issues as well as caregiving for their relatives. With the ongoing trend of nuclear families in rapidly urbanizing India, caring for their relatives residing in the rural areas became burdensome (Lukhmana et al., 2015).

Thus, FCs of the advanced cancer patients encounter psychological burden right from the time of cancer diagnosis of their relatives to even beyond the death of their relatives (Daya et al., 2018).

*Caregiving burden rate*

Unnikrishnan et al., did a study among FCs of both curative and palliative cancer patients in coastal south India and concluded that almost half of the caregivers had psychosocial burden, with 14% with moderate to severe levels of burden. (Unnikrishnan et al., 2019). Kong et al., studied burden among FCs of both inpatient and outpatient Malaysian cancer patients who were receiving predominantly (73.2%) active cancer therapy. They observed 55.6% caregiving burden rate among FCs of cancer patients (Kong et al., 2019). 

Whereas in our study, most of the FCs (51.65%) experienced moderate caregiving burden, 44.07% experienced high caregiving burden, and only 4.26% of FCs experienced mild caregiver’s burden. This higher caregiving burden may be due to enrolment of FCs of only advanced cancer patients in our study compared to both curative and palliative patients FCs enrolled in both Unnikrishnan et al., (2019)’s and Kong and Guan, (2019) studies.

Chakraborty et al., (2018) conducted a study among FCs of oral cancer patients receiving only palliative care services. They observed moderate to severe burden among 46 % of FCs, followed by mild to moderate burden in 36% of FCs. These results more or less corroborate with our study findings, suggesting generally higher caregiving burden among FCs of palliative care settings compared to curative settings. 

*Factors associated with caregiving burden*

Among the various demographic variables examined, the economic status of the FCs was the only factor found to be significantly associated with caregiving burden. Vashista et al., studied FCs of lung cancer patients from North India and observed significant reduction of quality of life (QOL) in caregivers residing in homes and earning less annual income per capita due to the added burden of caregiving (Vashista et al., 2019). This study findings corroborate with our study revealing significantly higher burden scores for FCs who belonged to BPL families (p=0.025). These findings highlighted the role of income in well-being of caregivers in developing countries, such as South Asian countries.

Vashista et al., also reported impaired QOL among rural caregivers population compared to urban population, but it was not lead to statistically significant difference. Similarly, we detected significantly higher caregiving burden among rural FCs. 

In contrast with Unnikrishnan et al., reporting that age of the caregivers was not found to be significantly associated with caregiving burden (p=0.291), we observed a significant relation between age of the caregivers and caregiving burden (p=0.039). 

*FCs satisfaction with palliative care*

Abernethy et al., showed that FCs had higher satisfaction with specialized palliative care provided to their relatives compared to general palliative care (Abernethy et al., 2007). 

Ullrich et al., conducted a pilot study on German FCs whose advanced cancer patients received specialized inpatient palliative care and observed that FCs satisfaction with these services was high based on scores obtained from FAMCARE-2 scale (73.4; SD 8.3). They also reported that the participants got higher scores in all subscales of FAMCARE-2 scale, despite the moderate to high caregiving burden of FCs (Ullrich et al., 2017). 

Oechsle et al, conducted a prospective multicentri trial on FCs of advanced inpatient cancer patients receiving specialized inpatient care and observed high satisfaction with palliative care with a mean total score of 73.7 on FAMCARE-2 scale(SD 9.6) (Oechsle et al., 2019). Similar findings were observed in our study, suggesting high satisfaction among FCs with our services.

*Factors associated with FCs satisfaction with palliative care*

Ullrich et al, found no associations between FCs satisfaction with patient care and caregiving burden (Ullrich et al., 2017). However, they observed higher satisfaction among female FCs with the palliative care services delivered. Oechsle et al, found no gender differences associated with the outcome. Our findings were similar to Oechsle et al.’ findings, revealing no association between gender and FAMCARE-2 score (Oechsle K et al, 2019). No significant association was observed between FC demographic characteristics and FC satisfaction.

This study was one of the first evaluating the specialized inpatient palliative care services through caregivers satisfaction living in South Asia. Given the prevalence of poor socio-economic conditions and inadequate formal caregiving services in South Asian countries like India and the findings of this study, the importance of interventions directed at relieving caregiving burden is highlighted. In addition, necessary measures to make widespread availability of dedicated inpatient palliative care services to advanced cancer patients in developing countries possible. 

*Limitations*

One of the limitations of this study was related to its cross-sectional nature. Cross-sectional studies assess functional outcomes at one single point in the course of a patient’s disease trajectory, but such stresses and satisfaction vary with time. Hence, longitudinal studies such as cohort studies or case control studies are suggested to establish the observed patterns in this study. Due to the high geographic and demographic variability in India and South Asia, our findings cannot be generalized. This issue can be addressed by conducting multi centric studies across India as well as whole South Asia. The potential confounding factors also included medical or psychiatric illness in FCs which could affect the caregiving burden outcomes.

In conclusion, to conclude, moderate to high caregiving burden was observed among FCs of advanced cancer patients receiving palliative care services in IPC in our study. We also observed significantly higher caregiving burden among the lower income FCs. These findings indicated the importance of regular assessment, finding the causal factors, and addressing these concerns through targeted interventions as early as possible. 

The FCs satisfaction with palliative care provided to their patients was high across all demographic groups, even though these groups showed moderate to high caregiving burden. With the regular employing of tools like FAMCARE-2 scale, we may assess both quantitative and qualitative functioning of palliative care services. Based on the assessments, we may introduce necessary interventions to achieve quality palliative care services to advance cancer patients.

## Author Contribution Statement

KAK and VSB designed the overall study. YA and RLN contributed to the conception, design, and conduct of the study with PSKC and PG acting as senior researchers overseeing the project. KAK and YA recruited study participants, collected data, and supported preparation of data. KAK and VSB prepared and supervised data collection, prepared data for statistical analyses, and conducted statistical analyses and literature search. KAK and VSB drafted the manuscript. All other authors provided comments and critical revisions. The final manuscript was approved by all authors prior to submission.
